# Treatment of experimentally induced partial-thickness burns in rats with different silver-impregnated dressings

**DOI:** 10.1590/acb370801

**Published:** 2022-11-28

**Authors:** Carolyna de Sousa Carvalho, Milton Junio Cândido Bernardes, Randys Caldeira Gonçalves, Marielle Sousa Vilela, Marcus Vinicius Meneses da Silva, Vinicius da Silva Oliveira, Marcelo Ribeiro da Rocha, Marina Clare Vinaud, Hélio Galdino, Ruy de Souza Lino

**Affiliations:** 1 MSc. Universidade Federal de Goiás – Tropical Pathology and Public Health Institute – Tropical Medicine and Public Health Graduation Program – Goiânia (GO), Brazil.; 2 PhD. Universidade Federal de Goiás – Tropical Pathology and Public Health Institute – Biology of the Host-Parasite Relationship Graduation Program – Goiânia (GO), Brazil.; 3 B.SC. Universidade Federal de Goiás – Nursing School – Goiânia (GO), Brazil.; 4 Graduate student. Universidade Federal de Goiás – Medicine School – Medicine Course – Goiânia (GO), Brazil.; 5 PhD. Universidade Federal de Goiás – Tropical Pathology and Public Health Institute – Biosciences Department – Goiânia (GO), Brazil.; 6 PhD. Universidade Federal de Goiás – Nursing School – Goiânia (GO), Brazil.; 7 PhD. Universidade Federal de Goiás – Tropical Pathology and Public Health Institute – Biosciences Department – Goiânia (GO), Brazil.

**Keywords:** Burns, Bandages, Animals, Acticoat, Collagen

## Abstract

**Purpose:**

To evaluate the morphometric, macroscopic and microscopic aspects of experimentally induced partial-thickness burns in rats treated with different silver-based dressings.

**Methods:**

Wistar rats were used, divided into six treatments: saline (NaCl 0.9%); silver sulfadiazine 1%; Silvercel; Mepilex Ag; Aquacel Ag and Acticoat. The animals were monitored daily and euthanized at 7, 14 and 30 days after injury induction (DAI).

**Results:**

At 7 DAI, necrosis/crust was greater in control, silver sulfadiazine and Mepilex Ag treatments, granulation tissue was induced by Aquacel Ag, polymorphonuclear infiltrate (PMN) infiltration was intensified by Mepilex Ag; mononuclear infiltrate (MN) infiltration and angiogenesis were increased by Silvercel. At 14 DAI, hemorrhage was decreased by Silvercel and Mepilex Ag, PMN infiltration increased by Acticoat. At 30 DAI, angiogenesis was greater in the Acticoat treatment and fibroblasts were increased by Acticoat and Mepilex Ag. Collagen was induced at 14 DAI by silver sulfadiazine and Aquacel Ag and, at 30 DAI, by silver sulfadiazine and Silvercel treatments.

**Conclusions:**

Silvercel and Acticoat presented better results than the other products. However, all the dressings were better than the control at some point during the process, and may contribute to the healing of partial thickness burns. Silvercel and Aquacel Ag treatments induced better cosmetic outcomes regarding wound closure and scarring.

## Introduction

Burns are recurrent traumatic injuries resulting from exposure to thermal, chemical, electrical or radioactive agents. Thermal agents are the causes of 86% of burns: electrical agents 4%, chemical agents 3% and other causes 7%. These lesions are classified according to their depth into: superficial thickness burns (1st degree) that affect only the skin epidermis; partial thickness burns (2nd degree) that affect the layers of the epidermis and dermis (superficial or deep) and full thickness burns (3rd degree) that extend to all layers of the skin (epidermis, dermis and subcutaneous tissue) causing total destruction of the tissue. These injuries are dynamic and may evolve over time, requiring, therefore, an accurate assessment by specialized professionals regarding the depth of the burn^1^,^2^.

Burns are considered a serious global health problem, which can lead to great morbidity and mortality. They are therefore a constant challenge for health professionals who deal with this type of traumatic injury daily, due to the severity of these injuries, which can vary depending on the extension, depth and body area involved and its multiple complications. In addition to the physical impact, this trauma can also cause serious psychological sequelae in individuals, directly affecting their quality of life, thus proper care and management of these patients is of paramount importance^1^,^3^,^4^.

Annually in Brazil, it is estimated that 1 million people are affected by burns, and 20% of those cases are treated in emergency units and only 5% are treated in specialized burn treatment centers^5^. The annual expenditure of the Brazilian Unified Health System with burn victims is approximately 15 million dollars^6^.

Currently, the most used topical product for this purpose is silver sulfadiazine introduced as the gold standard due to its antimicrobial and anti-inflammatory properties. However, it has some disadvantages, including delayed wound contracture, delayed and incomplete epithelialization, generation of black scars, limited penetration into the wound depth, hypersensitivity, neutropenia, silver toxicity, imprecise assessment of healing progress due to the presence of a peeling layer, ineffectiveness against some microorganisms and thrombocytopenia^7^–^9^.

Advances in wound care have resulted in the development of silver-based dressings. These therapies are considered new alternatives for the treatment of burns^10^–^12^. These dressings have antimicrobial properties due to the silver in their compositions which is released in a controlled and prolonged manner, has healing properties, in addition to being considered more practical, not requiring daily changes depending on the wound conditions, and can therefore reduce discomfort as well as lower patients’ recovery time^13^–^17^.

New therapies for the treatment of burn wounds can offer several benefits and in order to determine the effectiveness of these therapies and for selecting an efficient dressing it is essential to analyze the main pathological processes, associated with the phases of burn healing. Therefore, the aim of this study was to evaluate the morphometric, macroscopic and microscopic aspects of experimentally induced partial-thickness burns in rats, submitted to treatment with different silver-based dressings.

## Methods

This study was carried out at the Tropical Pathology and Public Health Institute (IPTSP), at the Universidade Federal de Goiás (UFG). The study was approved by the Ethics Committee on Animal Use, Protocol No. 84/2017. The animals were treated following the principles and guidelines of the National Council for the Control of Animal Experiments^18^.

### Experimentation animals

This was an experimental study using 90 female Wistar Hannover rats (*Rattus norvegicus* albinus), weighing approximately 200–250 g, from the Central Animal Facility, UFG.

Three rats were kept per cage. The cages used were made of polypropylene, lined with wood shavings, and the beds were changed twice a week. The animals received water and commercial autoclaved feed ad libitum. The luminosity, temperature, noise intensity and relative air humidity were those of the general environment.

### Experimental burn wound induction

On day 0 (zero), the animals were previously weighed and anesthetized by intraperitoneal administration of 10% ketamine (União Química Farmacêutica Nacional S/A, Brazil) and 2% xylazine, (Sespo Indústria e Comércio Ltd., Brazil), 0.1 mL/100 g of anesthetic solution (preparation: 0.6 mL of ketamine, 0.3 mL of xylazine and 0.1 mL of water injection). After the application of the anesthetic solution, the animal’s dorsal region was shaved, with subsequent antisepsis of the area to be burned, using sterile gauze soaked in a 70% alcohol solution. To perform the injury, the animal was placed inside a plastic cylinder made of polyvinyl chloride, with an opening of 2 × 2 cm^2^ and sealed ends. Then, a partial-thickness thermal injury was performed by immersing the exposed area of the animal’s back to boiling water at 95 °C for 7 s^19^–^21^.

### Postburn period

In the postburn period, the animals were kept with occlusive dressings, two per cage to avoid the opening of the dressings, thus minimizing the risk of possible trauma. The animals received analgesic medication: tramadol hydrochloride (Grünenthal do Brasil Farmacêutica Ltd.) to reduce pain, diluted in the drinking fountain, during the first 7 days after the induction of the lesion. Diet remained ad libitum.

### Debridement

On the second day after the induction of the lesions, the animals were weighed and anesthetized, using the same protocol as the burns, and underwent surgical debridement (tangential excision) according to The International Symposium on Biomedical Imaging^22^. To perform this procedure, a scalpel and scissors were used to remove the necrosis, and the skin was gently detached, preserving the subcutaneous muscle of the panniculus carnosus^20^,^21^.

### Experimental groups and products used

For the experimental procedure, the animals were randomly distributed into six groups: G1 control saline (NaCl 0.9%); G2 control treated with 1% silver sulfadiazine (Prati-Donaduzzi, Brazil, lot 16A273); Test G3 treated with Silvercel (Systagenix Brazil Import and commerce, lots 16804 and 25862); G4 test treated with Mepilex Ag (Molnlycke Health Care AB, lot 16231289); G5 test treated with Aquacel Ag (Convatec Incorp Limited, lot 403708); G6 test treated with Acticoat (Smith & Nephew Medical Limited, lot 66800396). All groups were monitored daily and euthanized at 7, 14 and 30 days after burn induction (DAI), using 5 rats per experimental day.

### Dressings

The animals in the control group (NaCl 0.9% solution) received daily, occlusive and sterilized dressings. Animals in the silver sulfadiazine group received a thin uniform layer of the product (1 mm), sufficient to cover the lesion bed^20^,^21^. The primary dressings of the animals, treated with Silvercel, Mepilex Ag and Acticoat were changed every 14 days, the Aquacel Ag was maintained until the end of the treatment, taking into account the manufacturer’s specifications. Only the secondary dressings were changed, the primary dressings were kept, in which the conditions and clinical aspects, such as dirt and humidity, were checked daily. Under sterile conditions, these dressings were cut into 2.5 × 2.5 cm^2^ squares before being applied to the burns. For the secondary dressing, sterile gauze and a calico cloth were used to protect the lesions.

### Dressing’s composition

Silver sulfadiazine: composed of cetostearyl alcohol, polyethylene glycol hexadecyl ether, liquid petrolatum, propylene glycol, methylparaben, propylparaben, butylhydroxytoluene and purified water. Silver-based dressings:

Silvercel: composed of nonwoven hydroalginate, calcium alginate, guluronic acid (high-G) strength of 32%, sodium carboxymethylcellulose (8%) and nylon fibers (51%) covered with elemental silver (9%).

Mepilex Ag: composed of a layer with silicone adhesives that stays in contact with the wound; absorbent polyurethane foam with a composite of silver and activated carbon; silver sulfate that releases silver ions; outer film permeable to water vapor and impervious to liquids.

Aquacel Ag: composed of sodium carboxymethylcellulose and silver; by two layers of hydrofiber, built in two-dimensional fibers; and gel.

Acticoat: composed of a layer of flexible, low-tack polyester coated with nanocrystalline silver; the silver level from maximum 1.64 mg/cm^2^ to 2.0 mg/cm^2^.

### Euthanasia

The animals were euthanized at the end of the experimental day (7, 14 and 30 DAI). The animals were individually placed inside a chamber with carbon dioxide (CO_2_) flow, following the recommendations of the *Guidelines for the Practice of Euthanasia of the National Council for Animal Experimentation*.

### Morphometric evaluations (wounds contraction)

In order to perform the morphometric analysis of wound contraction, the lesions were photographed using a digital camera attached to a tripod, at a constant distance of 11 cm, the images were recorded on day zero (burn wound induction day) and at the end of the experimental day (euthanasia day).

The delimitation of the burn area was performed using Image J software (National Institutes of Health, USA). To determine the degree of contraction of the burn area, Eq. 1, adapted from Moraes *et al*.^21^ was used:


$$\text{Relative wound contraction}(\%)=\frac{(\text{Initial injuried area}-\text{contracted injuried area)}}{\text{Initial injuried area}}\times 100$$ (1)

### Macroscopic evaluations

On the established experimental days, the phases of the healing process (inflammatory, proliferative and remodeling phases) were macroscopically analyzed. The following parameters were evaluated: necrosis/crust, granulation tissue and reepithelialization, identified in a semiquantitative way, according to the following criteria: absent (score 0); discreet (score 1 – up to 25% of compromised area); moderate (score 2 – between 26 and 50% of compromised area) and severe (score 3 – above 50% of compromised area)^20^.

### Microscopic evaluation

The microscopic evaluation was performed from wound fragments removed by biopsy and fixed in 10% buffered formaldehyde (pH 7.2). Subsequently, this material was processed for inclusion in paraffin. The paraffin blocks were placed in a microtome (Leica RM2255), serial sections of the material (4 μm) were obtained and placed on glass slides. The slides were stained with hematoxylin and eosin (H&E) and picrosirius red (PS).

The analysis of general pathological processes was performed in the H&E-stained slides and were evaluated using a binocular microscope (Leica DM750), coupled to a camera (Leica ICC50 HD) to record the images. The presence of the following parameters was analyzed: necrosis/crust, hemorrhage, fibrin, polymorphonuclear infiltrate (PMN), mononuclear infiltrate (MN), angiogenesis and fibroblasts, the entire extent of the wound was evaluated. At 30 DAI, wound closure was analyzed. These parameters were identified in a semiquantitative way, following the criteria of Fantinati *et al*.^20^, described previously.

The PS-stained slides were used for the collagen quantification. They were evaluated in a binocular microscope (Zeiss Axiostar Plus) and recorded with a digital camera (Sony Alpha Nex-3). Collagen fibers were evaluated under polarized light, and the entire length of the slides were visualized and photographed. For this analysis, the Image J software (National Institutes of Health, USA) was used.

### Statistical analysis

Statistical analysis was performed using Sigma Stat 2.3 software. All variables were tested for normal distribution and homogeneous variance. For the morphometric analysis, the parametric ANOVA test and Tukey’s post-test were used. For macroscopic and microscopic analyses, the nonparametric Kruskal–Wallis test and Dunn’s post-test were used. For analysis of wound closure and contraction, Fisher’s exact test was used. The observed differences were considered significant when p < 0.05.

## Results

There were no animal losses during the experimental period of this study. The pathologic processes were evaluated regarding the following parameters: contraction rate (morphometric analysis), macroscopic and microscopic analyses which enabled the characterization of the phases of the inflammatory process, in other words, inflammatory (7 DAI), proliferative (14 DAI) and remodeling (30 DAI).

### Morphometric analysis

The contraction degree of the burn wounds at 14 DAI was significantly higher after the Mepilex Ag treatment of the wound in comparison to the control group (p < 0.007), while the other treatments did not interfere with the wound contraction.

### Macroscopic analysis

When comparing the treatments with the control groups (NaCl 0.9% and silver sulfadiazine), in the inflammatory phase (7 DAI), there was less necrosis/crust in the wounds after the Silvercel and Aquacel Ag treatment (p < 0.05).

On the other hand, the comparison between the treatments showed that Aquacel Ag presented less necrosis/crust than Silvercel (p < 0.05). The treatment with Mepilex Ag induced greater necrosis/crust in comparison to the Silvercel, Aquacel Ag and Acticoat treatments (p < 0.05).

**Figure 1:**
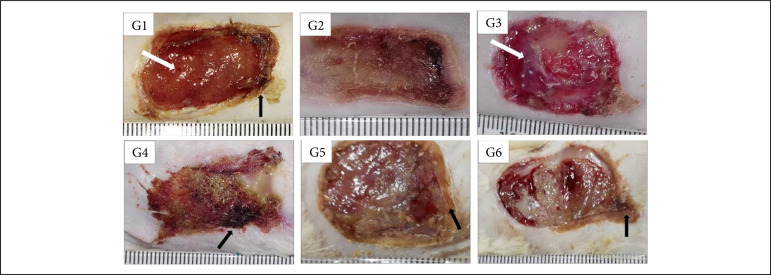
Macroscopic aspects of partial thickness burn wounds experimentally induced in Wistar rats treated with silver-based dressings through 7 days.

**Figure 2:**
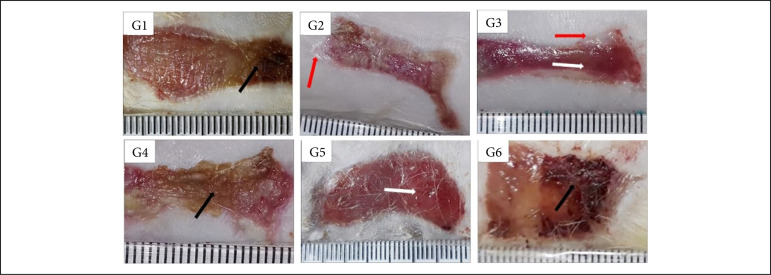
Macroscopic aspects of partial thickness burn wounds experimentally induced in Wistar rats treated with silver-based dressings through 14 days.

**Figure 3:**
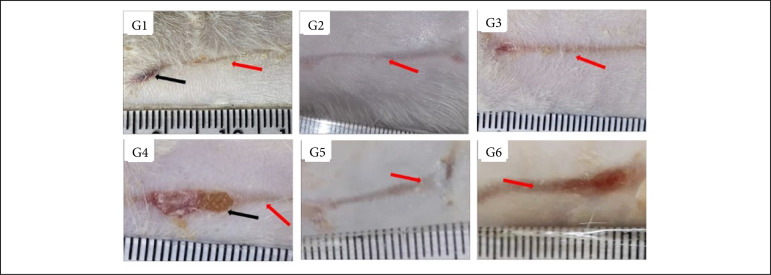
Macroscopic aspects of partial thickness burn wounds experimentally induced in Wistar rats treated with silver-based dressings through 30 days.

Regarding the granulation tissue, the comparison between the treatments and the control groups, at 7 DAI, showed that the group treated with Aquacel Ag presented greater granulation tissue than the one treated with silver sulfadiazine (p < 0.05). The comparison between treatments allowed the observation that Silvercel induced greater granulation tissue (p < 0.05) in comparison to Mepilex Ag and the wounds treated with Aquacel Ag presented greater granulation tissue than the ones treated with Mepilex Ag and Acticoat (Table 1, Fig. 1). During the proliferative phase (14 DAI) Silvercel and Aquacel Ag treatments induced less necrosis/crust than the Mepilex Ag treatment (p < 0.05) (Table 1, Fig. 2). In the remodeling phase (30 DAI), the treatments with Silvercel, Aquacel Ag and Acticoat induced lower necrosis/crust (p < 0.05) in comparison to the control. On the other hand, the wounds treated with Mepilex Ag presented greater necrosis/crust and less reepithelialization (p < 0.05) in comparison to the wounds treated with silver sulphadiazine, Silvercel, Aquacel Ag and Acticoat (Table 1, Fig. 3).

In Figs. 1 to 12, and Tables 1 to 4 : G1 – control group treated with NaCl 0.9%; G2 – group treated with silver sulphadiazine 1%; G3 – group treated with Silvercel; G4 – group treated with Mepilex Ag; G5 – group treated with Aquacel Ag; G6 – group treated with Acticoat.

**Table 1:** Macroscopic analysis of the general pathologic processes in burn wounds experimentally inducedin Wistar rats and treated with silver-based dressings.

Pathologic process	DAI	G1 (n=15) Median (min-max)	G2 (n=15) Median (min-max)	G3 (n=15) Median (min-max)	G4 (n=15) Median (min-max)	G5 (n=15) Median (min-max)	G6 (n=15) Median (min-max)	p	Dunn’s
Necrosis/crust	7	(2-3)	3 (2-3)	2 (1-2)	3 (3-3)	0.5 (0-1)	1 (1-3)	0.001*	G1>G3; G1>G5; G2>G3; G2>G5; G3>G5; G4>G3 G4>G5; G4>G6; G6>G5
	14	2 (1-3)	3 (0-3)	1 (0-2)	2 (2-3)	0 (0-1)	1 (0-3)	0.010*	G4>G3; G4>G5
	30	2 (0-3)	0.5 (0-2)	0 (0-1)	2.5 (0-3)	0 (0-1)	0 (0-1)	0.008*	G1>G3; G1>G5; G1>G6; G4>G2; G4>G3; G4>G5; G4>G6
Granulation tissue	7	2 (1-3)	1 (1-2)	2 (1-3)	1 (0-1)	3 (3-3)	1 (0-3)	0.001*	G3>G4; G5>G1; G5>G2; G5>G4; G5>G6
	14	2.5 (2-3)	2 (1-3)	3 (2-3)	2 (1-3)	3 (2-3)	3 (1-3)	0.378	
	30	0 (0-0)	0 (0-1)	0 (0-1)	0 (0-0)	0 (0-1)	1 (0-1)	0.07	
Reepithelialization	7	0 (0-0)	0 (0-0)	0 (0-0)	0 (0-0)	0 (0-0)	0 (0-0)	1.000	
	14	0.5 (0-2)	0 (0-2)	0 (0-2)	2 (0-2)	2 (2-2)	1 (0-1)	0.157	
	30	3 (2-3)	3 (3-3)	3 (3-3)	2 (2-3)	3 (3-3)	3 (3-3)	0.012*	G2>G4; G3>G4; G5>G4; G6>G4

DAI – days after injury induction; n – number of animals per group; Min - minimum value; max – maximum value. Statistical analysis: Kruskal-Wallis followed by Dunn`s post-test.

* - statistical significance.

Black arrow – necrosis/crust. White arrow: granulation tissue. Scale in mm.

Black arrow – necrosis/crust. White arrow: granulation tissue. Red arrow: reepithelialization. Scale in mm.

Black arrow – necrosis/crust. Red arrow: reepithelialization. Scale in mm.

### Microscopic analysis

**Figure 4:**
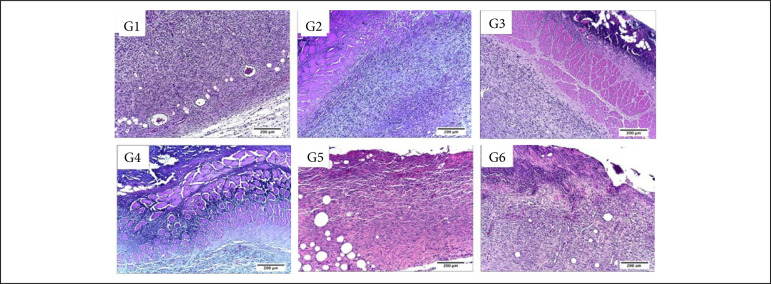
Microscopic aspects of partial thickness burn wounds experimentally induced in Wistar rats and treated with silver-based dressings 7 DAI, scale bar = 200 μm.

**Figure 5:**
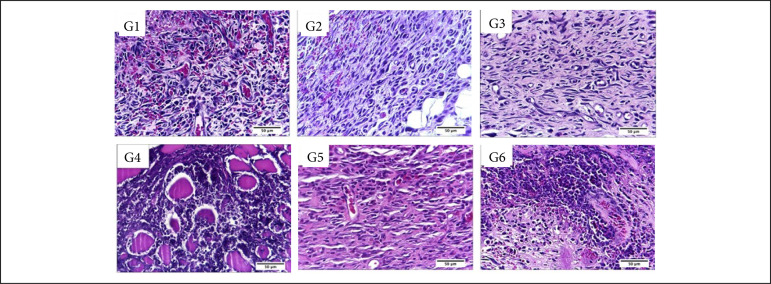
Microscopic aspects of partial thickness burn wounds experimentally induced in Wistar rats and treated with silver-based dressings 7 DAI, scale bar = 50 μm.

In the inflammatory phase (7 DAI) regarding the presence of necrosis/crust the comparison between the treated and control groups showed that the group treated with Mepilex Ag and Acticoat presented greater necrosis/crust (p < 0.05) in comparison to the control groups. Also, the group treated with Acticoat presented greater necrosis/crust than all the other treatments (p < 0.05) (Table 2, Figs. 4 and 5).

**Table 2:** Microscopic analysis of the general pathologic processes in partial thickness burn wounds experimentally induced in Wistar rats and treated with silver-based dressings.

Pathologic process	DAI	G1 (n=15) Median (min-max)	G2 (n=15) Median (min-max)	G3 (n=15) Median (min-max)	G4 (n=15) Median (min-max)	G5 (n=15) Median (min-max)	G6 (n=15) Median (min-max)	p	Dunn’s
Necrosis/Crust	7	2 (1-3)	1 (1-2)	2 (1-2)	3 (3-3)	2 (2-2)	2(2-3)	0.001*	G4>G1; G4>G2; G4>G3; G4>G5; G6>G2
	14	1 (1-1)	2 (1-3)	1 (1-1)	2 (1-2)	1(1-2)	2(1-2)	0.025*	G2>G1; G2>G3; G4>G1; G4>G3; G6>G1; G6>G3
	30	0 (0-1)	1 (0-1)	0 (0-1)	0 (0-1)	0(0-1)	0(0-1)	0.595	
Hemorrhage	7	2 (0-3)	2 (1-2)	2 (1-3)	1 (1-2)	1(0-2)	2(1-3)	0.300	
	14	0 (0-0)	1 (1-2)	0 (0-0)	0 (0-0)	1 (0-2)	2 (0-2)	0.001*	G2>G1; G2>G3; G2>G4; G5>G1; G5>G3; G5>G4; G6>G1; G6>G3; G6>G4;
	30	0 (0-0)	0 (0-0)	0 (0-0)	0 (0-0)	0 (0-0)	0 (0-0)	1.000	
Fibrin	7	1 (1-3)	2 (2-3)	1 (1-1)	1 (1-2)	2 (1-3)	2 (1-3)	0.017*	G2>G3
	14	1 (0-1)	1 (1-2)	0 (0-0)	0 (0-0)	0 (0-1)	0 (0-1)	0.002*	G2>G3; G2>G4; G2>G5; G2>G6
	30	0 (0-0)	0 (0-0)	0 (0-0)	0 (0-0)	0 (-0-0)	0 (0-0)	1.000	
PMN	7	2 (1-3)	1 (1-2)	1,5 (1-2)	3 (3-3)	2 (1-2)	2 (2-3)	0.001*	G4>G1; G4>G2; G4>G3; G4>G5; G4>G6
	14	1 (1-1)	2 (1-3)	1 (1-1)	2 (1-2)	1 (1-3)	2 (1-3)	0.016*	G6>G1; G6>G3
	30	0 (0-1)	1 (0-1)	0 (0-1)	0 (0-1)	1 (0-1)	0 (0-1)	0.417	
MN	7	2 (2-3)	3 (3-3)	3 (3-3)	2 (2-3)	2 (1-2)	1 (1-2)	0.001*	G1>G5; G1>G6; G2>G4; G2>G5; G2>G6; G3>G4; G3>G5; G3>G6; G4>G6
	14	2 (2-3)	2 (2-2)	2 (2-2)	2 (2-2)	2 (2-3)	2 (2-3)	0.153	
	30	1 (1-2)	2 (1-2)	1 (1-1)	2 (1-3)	1 (1-2)	1 (1-2)	0.106	
Angiogenesis	7	3 (2-3)	3 (3-3)	3 (3-3)	2 (2-3)	3 (1-3)	2 (2-3)	0.002*	G2>G4; G2>G6; G3>G4; G3>G6
	14	3 (2-3)	2 (2-2)	3 (3-3)	3 (2-3)	3 (3-3)	3 (3-3)	0.001*	G1>G2; G3>G2; G4>G2; G5>G2; G6>G2
	30	1 (1-1)	1 (1-2)	1 (1-2)	1 (1-2)	2 (1-3)	2 (2-3)	0.011*	G6>G1; G6>G2; G6>G3; G6>G4
Fibroblast	7	3 (3-3)	3 (3-3)	3 (3-3)	3 (3-3)	2 (1-2)	1 (1-3)	0.001*	G1>G5; G1>G6; G2>G5; G2>G6; G3>G5; G3>G6; G4>G5; G4>G6
	14	3 (3-3)	3 (3-3)	3 (3-3)	3 (3-3)	3 (3-3)	3 (3-3)	1.000	
	30	2 (2-3)	2 (2-2)	2 (2-2)	3 (2-3)	2 (1-3)	3 (2-3)	0.035*	G4>G2; G4>G3; G4>G5; G6>G2; G6>G3; G6>G5

DAI – days after injury induction; n – number of animals per group; min – minimum value; max – maximum value. PMN – polymorphonuclear cells infiltration; MN – mononuclear cells infiltration. For the statistical analysis the following criteria were considered: 0 – absent; 1 – discrete; 2 – moderate; 3 – accentuated. Statistical tests used: Kruskal-Wallis followed by Dunn`s post-test.

* - statistical significance.

The Silvercel treated group presented significantly more fibrin than what was observed after the silver sulphadiazine treatment (p < 0.05). The PMN inflammatory infiltration was significantly more intense after the Mepilex Ag treatment in comparison to all the other treatments (p < 0.05).

While the MN inflammatory infiltration was more intense in the control group, treated with NaCl 0.9%, in comparison to the Aquacel Ag and Acticoat treatments (p < 0.05). The silver sulphadiazine and Silvercel also induced greater MN infiltration than the Mepilex Ag, Aquacel Ag and Acticoat ones (p < 0.05).

Angiogenesis was significantly more intense in the silver sulphadiazine and in the Silvercel treated groups in comparison to Mepilex Ag and Acticoat ones (p < 0.05). Fibroblasts were significantly more observed in the control, silver sulphadiazine, Silvercel and Mepilex Ag treated groups in comparison to Aquacel Ag and Acticoat groups (p < 0.05) (Table 2, Figs. 4 and 5).

**Figure 6:**
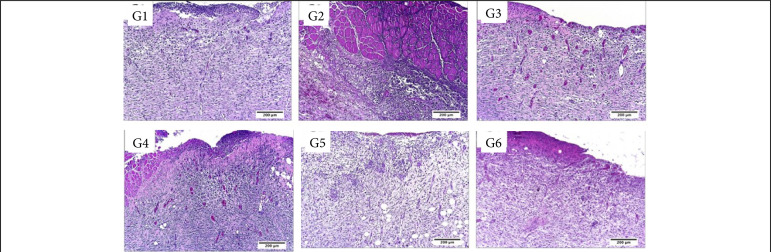
Microscopic aspects of partial thickness burn wounds experimentally induced in Wistar rats and treated with silver-based dressings 14 DAI, scale bar = 200 μm.

**Figure 7:**
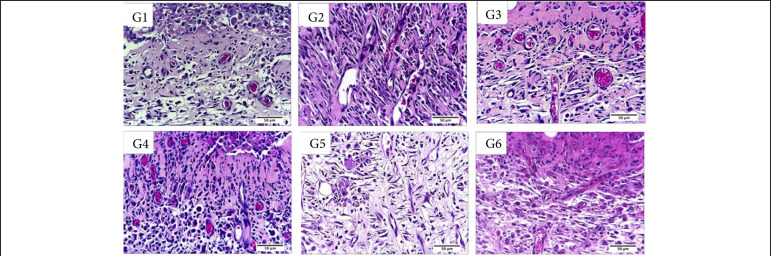
Microscopic aspects of partial thickness burn wounds experimentally induced in Wistar rats and treated with silver-based dressings 14 DAI, scale bar = 50 μm.

While in the proliferative phase, 14 DAI, there was significantly more necrosis/crust in the silver sulphadiazine, Mepilex Ag and Acticoat treated groups in comparison to the control and Silvercel treated groups (p < 0.05). There was significantly more hemorrhage in the silver sulphadiazine, Aquacel Ag and Acticoat treated groups in comparison to the control, Silvercel and Mepilex Ag ones (p < 0.05). Fibrin was observed more intensely in the silver sulphadiazine treated group in comparison to all the other treatments (p < 0.05). The PMN infiltration was more intense in the Acticoat treated group than in the control and Silvercel groups (p < 0.05). While the silver sulphadiazine group presented less hemorrhage than all the other treatments (p < 0.05) (Table 2, Figs. 6 and 7).

**Figure 8:**
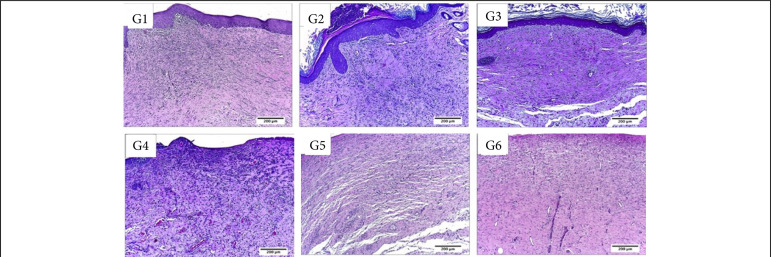
Microscopic aspects of partial thickness burn wounds experimentally induced in Wistar rats and treated with silver-based dressings 30 DAI, scale bar = 200 μm.

**Figure 9:**
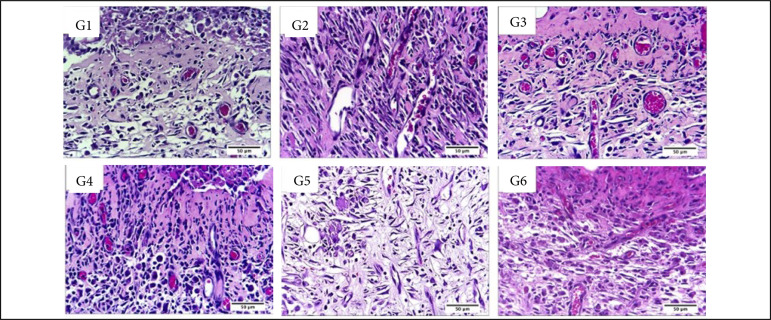
Microscopic aspects of partial thickness burn wounds experimentally induced in Wistar rats and treated with silver-based dressings 7 DAI, scale bar = 50 μm.

In the remodeling phase, 30 DAI, was possible to observe greater angiogenesis in the Acticoat treated group in comparison to all the other treatments. There was more fibrin in the Mepilex Ag and Acticoat treatments in comparison to silver sulphadiazine, Silvercel and Aquacel Ag treatments (p < 0.05) (Table 2, Figs. 8 and 9).

There was greater wound closure in the groups treated with NaCl 0.9% (control), silver sulphadiazine, Silvercel and Mepilex Ag in comparison to the other treatments (p < 0.05) (Table 3).

**Table 3:** Wound closure 30 DAI in partial thickness burn wounds experimentally induced in Wistar ratsand treated with silver-based dressings.

Wound closure	G1	G2	G3	G4	G5	G6	p
Yes	4	4	5	2	1	1	1.00
No	1	1	0	3	4	4	
Total	5	5	5	5	5	5	

Statistical tests used: Fischer.

### Collagen fibers quantification

**Figure 10:**
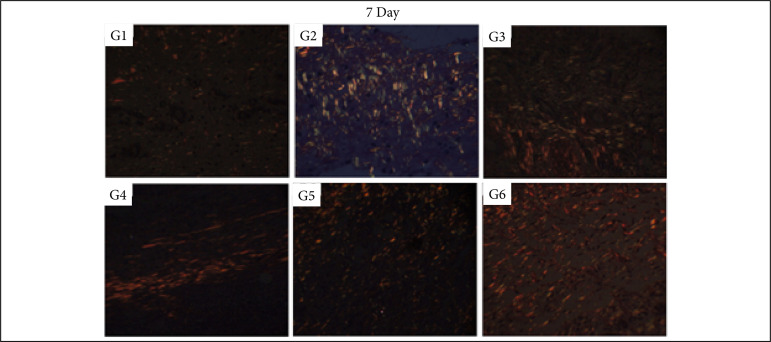
Collagen fibers deposition in partial thickness burn wounds experimentally induced in Wistar rats and treated with silver-based dressings 7 DAI. Type I collagen fibers are shown in red and type III collagen fibers are shown in green.

In the inflammatory phase (7 DAI) of the healing process, there was significantly more collagen fibers deposition after the treatment with silver sulphadiazine in comparison to control (NaCl 0.9%), Silvercel, Mepilex Ag and Aquacel Ag treatments (p < 0.05). The Silvercel treatment induced greater collagen deposition in comparison to the control and Mepilex ones. The Aquacel Ag and Acticoat treatments showed greater collagen deposition in comparison to control and Mepilex Ag ones (p < 0.05). Therefore, the greater amount of collagen fibers was observed sequentially after the treatment with silver sulfadiazine, Silvercel, Aquacel Ag and Acticoat (Table 4, Fig. 10).

**Figure 11:**
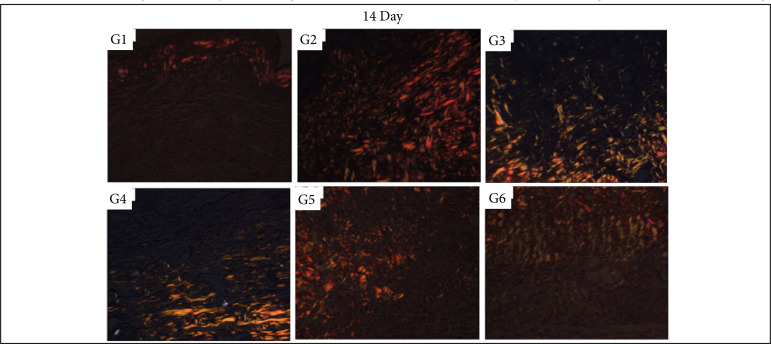
Collagen fibers deposition in partial thickness burn wounds experimentally induced in Wistar rats and treated with silver-based dressings 14 DAI. Type I collagen fibers are shown in red and type III collagen fibers are shown in green.

In the proliferative phase (14 DAI) it was possible to observe more collagen fibers deposition in the silver sulphadiazine treatment in comparison to all other ones (p<0.05). While the Silvercel, Mepilex Ag, Aquacel Ag and Acticoat showed greater collagen fibers deposition than the control group (p<0.05). Therefore, sequentially there was more collagen fibers after the treatment with silver sulfadiazine and Aquacel Ag (Table 4, Fig. 11).

**Figure 12:**
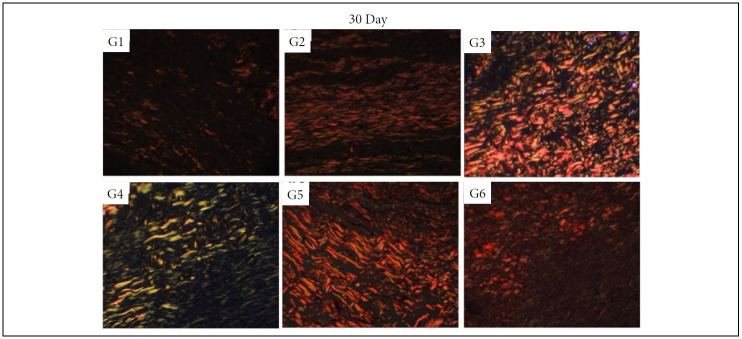
Collagen fibers deposition in partial thickness burn wounds experimentally induced in Wistar rats and treated with silver-based dressings 30 DAI. Type I collagen fibers are shown in red and type III collagen fibers areshown in green.

In the remodeling phase (30 DAI) all treatments presented greater collagen deposition than the control group (p < 0.05). While the Acticoat treatment induced less collagen deposition than the other treatments (p < 0.05) (Table 4, Fig. 12).

**Table 4:** Quantitative analysis of collagen fibers deposition in partial thickness burn woundsexperimentally induced in Wistar rats and treated with silver-based dressings.

DAI	G1 (n=15) Median (min-max)	G2 (n=15) Median (min-max)	G3 (n=15) Median (min-max)	G4 (n=15) Median (min-max)	G5 (n=15) Median (min-max)	G6 (n=15) Median (min-max)	p	Dunn’s
7	0.03 (0.00-1.51)	0.73 (0.00-9.55)	0.42 (0.00-9.90)	0.07 (0.00-0.19)	0.29 (0.02-9.69)	0.61 (0.02-7.31)	0.001*	G2>G1; G2>G3; G2>G4; G2>G5; G3>G1; G3>G4; G5>G1; G5>G4; G6>G1; G6>G4
14	0.02 (0.00-5.02)	1.79 (0.02-15.62)	0.25 (0.06-4.98)	0.30 (0.02-6.37)	0.60 (0.03-9.80)	0.24 (0.05-5.93)	0.001*	G2>G1; G2>G3; G2>G4; G2>G5; G2>G6; G3>G1; G4>G1; G5>G1; G5>G6; G6>G1
30	0.02 (0.00-4.25)	4.26 (0.12-18.73)	18.01 (1.17-57.00)	9.15 (1.36-46.67)	7.61 (0.26-45.70)	1.52 (0.12-36.61)	0.001*	G2>G1; G3>G1; G3>G6; G4>G1; G4>G6; G5>G1; G5>G6; G6>G1

DAI – days after injury induction; n – number of animals per group; min – minimum value; max – maximum value. Statistical tests used: Kruskal-Wallis followed by Dunn`s post-test.

* - statistical significance.

Stain: Picrosirius Red. Scale in μm. Augmentation 20×.

Stain: Picrosirius Red. Scale in μm. Augmentation 20×.

Stain: Picrosirius Red. Scale in μm. Augmentation 20×.

## Discussion

This study evaluated the dynamic of the healing phases of partial thickness burn wounds treated with different silver-based products showing the different effects observed throughout 30 days of follow-up. It is interesting to highlight that at each phase of the healing process a different product showed better qualities and results regarding the closure of the wound, the inflammatory reaction, the collagen production and the scar formation. These findings reinforce that there is no consensus on the ideal burn dressing and that several microscopic and macroscopic aspects should be taken under consideration^23^. It is important to highlight that the findings described in this study reflect the animal’s response to the treatments performed during the experimental period of 30 days.

The products Silvercel and Aquacel Ag are the only ones that have carboxymethylcellulose in their composition, which is an anionic polymer, capable of binding to metals (silver), enhancing its reactivity with organic matter. This can generate positive results in the inflammatory phase (first 7 days of the healing process) such as less necrosis/crust formation when compared to the control group and the group treated with silver sulfadiazine. In addition, Aquacel Ag showed less necrosis than the ones observed in lesions treated with Silvercel, Mepilex Ag and Acticoat. The hydrofiber present in the Aquacel Ag composition can to absorb the wound exudate, which when associated with sodium carboxymethylcellulose contributes to the role of hydrating the wound bed. Therefore, Aquacel Ag can absorb the wound exudate and form a gel, thereby decreasing the formation of necrosis/crust^24^,^25^. The presence and formation of necrosis/crust in the wound bed impairs the healing process as it prevents cell migration and, consequently, the re-epithelialization of the lesion; interferes with the formation of granulation tissue and enables colonization by microorganisms, thus prolonging the inflammatory phase of healing^26^–^28^.

Silvercel was evaluated in the treatment of pressure wounds and after 7 days induced a decrease in necrosis, less exudate, odor, and increased the formation of granulation tissue with good evolution throughout the evaluated period^9^,^29^ which is in accordance with the results of this study. The lower necrosis/crust observed in the Silvercel treated wounds may have occurred due to presence of hydroalginate and calcium alginate in its composition which promotes the exchange of calcium ions for sodium ions and induces a better hydration of the wound bed and contributes to the autolytic debridement^15^,^30^–^32^.

Mepilex Ag is considered an advanced wound dressing that is designed in order to be replaced with less frequency promoting humidity in the wound bed without the necrosis/crust formation as observed when used in the treatment of diabetic foot ulcers^25^, pressure wounds^33^ and pediatric burn wounds^34^, which is in accordance to these findings in the treatment of experimental partial-thickness burn wounds.

The absence of hydrogel in the composition of Mepilex Ag and Acticoat, which is essential for the moisturizing of the wound bed, may explain the greater formation of necrosis/crust in the wounds treated with both products in our study. However, Acticoat presented a significant increase in re-epithelialization of burn wounds, reduced use of antibiotics due to the graduate silver release during the application of the dressing and was related to the reduction of the overall healing time, wound exudate, less need for surgical procedures such as grafting, less hypertrophic scarring and infection rates^35^.

On the other hand, Mepilex Ag presented increase in reepithelialization of burn wounds which were observed more quickly after this treatment in comparison to other products such as Acticoat and Aquacel Ag in a randomized trial study of the healing process in partial thickness burn wounds^23^. Mepilex Ag presents a foam in its composition which is responsible for absorbing the wound exudate which leaves the wound bed less hydrated and contributes to the necrosis/crust formation^13^.

Macroscopically, Aquacel Ag induced greater granulation tissue in the inflammatory phase of the healing process (7 DAI), which is considered a good quality of this product because it shows induction of angiogenesis and greater migration of cells such as fibroblasts and indicate progress in the healing process^36^.

Microscopically, in the proliferative phase of the healing process (14 DAI), the silver sulfadiazine, Mepilex Ag and Acticoat treatments induced greater necrosis/crust in comparison to the other treatments. Silver sulfadiazine is the most used antimicrobial and healing topic substances in the treatment of burn, surgical, ulcers and pressure wounds^4^,^15^. This product does not present in its composition anything that promotes the hydration of the wound bed which contributes to the greater formation of necrosis/crust during the first two weeks of the healing process^35^,^37^,^38^.

Regarding the inflammatory infiltration of PMN observed greatly in the inflammatory phase of the healing process (7 DAI), Mepilex Ag induced greater infiltration than the other treatments, followed by Acticoat. While the MN infiltration was greatly induced by Silvercel and silver sulfadiazine. The presence of inflammatory cells is crucial in the first days of the healing process to prevent the proliferation of microorganisms and to eliminate foreign body particles and necrotic debris. Also, these cells are responsible for the production and liberation of inflammatory mediators such as TGF-β, EGF, TNF-α and VEGF amongst others are important in the transition from the inflammatory phase of the healing process to the proliferative one^27^,^36^.

In the matter of the proliferative phase of the healing process (14 DAI), there was less hemorrhage and more angiogenesis in the wounds treated with Mepilex Ag and Silvercel in comparison to the control groups and to the other treatments. The increase in angiogenesis is crucial to provide adequate oxygenation and nutrition for the new tissue on the wound bed contributing to the formation of granulation tissue^29^,^39^. Silvercel presents the ability to absorb the wound exudate diminishing the bleeding and increasing vasodilation which favors angiogenesis and is positive regarding the whole healing process^29^,^39^. The increase in hemorrhage leads to higher fibrin in the wounds which is a response to endothelial production of eicosanoids and leukotrienes which in turn contributes to a progressive increase in the vascular permeability to migrant cells and biologically active substances^27^,^40^–^42^.

In the remodeling phase of the healing process (30 DAI) there was an increase in angiogenesis in the Acticoat treated group and in fibroblasts in the Mepilex Ag and Acticoat treated groups. These are important effects that contribute to the complete wound closure and collagen formation. The increase in fibroblast in the wound bed is important to the synthesis of collagen, elastin, glycoproteins and proteoglycans of the extracellular matrix which in turn increases the tensile strength of the edges of the lesion resulting in the wound closure^43^. Wet dressings containing hydrogel, alginate and/or hydrocolloids have been stimulated in the treatment of burn wounds because of the moisture suppressing scar formation, the stimulus of keratinocyte migration and reepithelialization. Also, these dressings improve collagen formation contributing to deposition and organization of newly formed collagen fibers^44^.

The presence of silver in all dressings used in this study is a factor that accelerates the healing process due to the reduction of secondary infections on the wound. Also, the diminished number of changes of the dressings decrease the wound manipulation which also contributes to the healing process^25^,^45^.

This study may contribute to the elaboration of a protocol in handling burn wounds with special concern regarding the easier handling of the patients with less pain. The public health system in Brazil may greatly benefit from this study since it may lead to diminished costs in the treatment of burn wounds due to less contamination of dressings when there is the use of silver-based dressings and quicker recovery with less scars. The need of consensus protocol for the treatment of burn wounds in Brazil has been reviewed by Fuculo-Junior *et al*.^46^.

## Conclusion

Considering all aspects of the healing process analyzed in this study using the murine model of partial thickness burn wounds, Silvercel and Acticoat presented better results than the other products. However, all the dressings analyzed were better than the control treatment with NaCl 0.9% at some point during the process, and may contribute to the healing of partial thickness burns. These results have the potential to strengthen the indications for these silver-based dressings, as the wounds are dynamic and it depends on the professional to recognize each phase in order to select the best therapeutic option. Silvercel and Aquacel Ag treatments induced better cosmetic outcome regarding wound closure and scarring.
